# Theory and practice of integrative clinical ethics support: a joint experience within gender affirmative care

**DOI:** 10.1186/s12910-020-00520-3

**Published:** 2020-08-26

**Authors:** Laura Hartman, Giulia Inguaggiato, Guy Widdershoven, Annelijn Wensing-Kruger, Bert Molewijk

**Affiliations:** 1 Department of Ethics, Law and Humanities, Amsterdam UMC, Vrije Universitieit Amsterda, Amsterdam, The Netherlands; 2 Centre of Expertise on Gender Dysphoria, Amsterdam UMC, Vrije Universiteit Amsterdam, Amsterdam, The Netherlands; 3 Department of Medical Psychology, Amsterdam UMC, Vrije Universiteit Amsterdam, Amsterdam, The Netherlands; 4 Centre for Medical Ethics, Institute of Health and Society, Faculty of Medicine, University of Oslo, Oslo, Norway

**Keywords:** Clinical ethics support, Theory, Pragmatism, Hermeneutics, Gender affirmative care, Integrative, Responsive evaluation

## Abstract

**Background:**

Clinical ethics support (CES) aims to support health care professionals in dealing with ethical issues in clinical practice. Although the prevalence of CES is increasing, it does meet challenges and pressing questions regarding implementation and organization. In this paper we present a specific way of organizing CES, which we have called integrative CES, and argue that this approach meets some of the challenges regarding implementation and organization.

**Methods:**

This integrative approach was developed in an iterative process, combining actual experiences in a case study in which we offered CES to a team that provides transgender health care and reflecting on the theoretical underpinnings of our work stemming from pragmatism, hermeneutics and organizational and educational sciences.

**Results:**

In this paper we describe five key characteristics of an integrative approach to CES; 1. Positioning CES more within care practices, 2. Involving new perspectives, 3. Creating co-ownership of CES, 4. Paying attention to follow up, and 5. Developing innovative CES activities through an emerging design.

**Conclusions:**

In the discussion we compare this approach to the integrated approach to CES developed in the US and the hub and spokes strategy developed in Canada. Furthermore, we reflect on how an integrative approach to CES can help to handle some of the challenges of current CES.

## Background

Clinical ethics support (CES) aims to support health care professionals in dealing with ethical issues in clinical practice. Since the seventies of the last century the prevalence of CES is gradually increasing in Europe, the US and Canada [11]. Regulatory agencies, who issue health care organizations their accreditation, also increasingly mention the importance of CES services for both health care professionals and the quality of health care [23, 24]. CES is provided by different services (e.g. ethics committee, ethics consultation, moral case deliberation (MCD)) with varying aims, methods and procedures [11, 30].

Although the prevalence of CES is increasing, it does meet challenges and pressing questions regarding implementation and organization [12, 39, 46]. Research shows, that CES providers encounter difficulties in setting up the collaboration with clinicians, in receiving cases, and in following the uptake of their advice [29, 31, 40]. Also, there is uncertainty about the role of the CES professional, and discussion whether the CES professional should take an insider or an outsider position with regards to the team or health care organization. Should the CES professional be independent, and have the role of critically highlighting moral issues or should the CES professional have a position in the team or health care organization [39]?

In this paper we present a specific way of organizing CES, which we have called integrative CES, and argue that this approach meets some of the challenges regarding implementation and organization. This integrative approach was developed in an iterative process, by combining the insights we gained from our experience in a case study in which we offered CES to a team of professionals (see methods) with the reflection on the theoretical underpinnings of our work. We did not have a blue-print beforehand on what integrative CES could entail. Below, we describe what we mean by integrative CES by elaborating on five key characteristics; 1. Positioning CES more within care practices, 2. Involving new perspectives, 3. Creating co-ownership of CES, 4. Paying attention to follow up, and 5. Developing innovative CES activities through an emerging design.

In the following, we first outline the theoretical background of our approach to CES in general. Then, we describe the five key characteristics of Integrative CES. In the discussion we compare this approach to the integrated approach to CES developed in the US and the hub and spokes strategy developed in Canada. Finally, we reflect on whether the approach described here can meet some of the challenges of current CES and shed new light on the role of the CES professional and the distinction between insider and outsider position.

## Methods

### Theoretical framework

Our approach to clinical ethics is theoretically grounded in hermeneutic ethics and pragmatism [21, 47, 48]. This implies a specific understanding of *morality* and *ethics.* We understand *morality* as the complex background of one’s education, culture, professional norms, family, peer network etc. One acquires this background in an implicit way; by moving through the world, a person finds out what works, what doesn’t work, learns new skills and picks up what is valued in one’s family or organizational culture. This moral framework is usually invisible and not reflected upon. It is the framework from which a person perceives the world. This moral framework has also been characterized in terms of moral routines or habits [10, 25, 32].

We understand *ethics* as the reflection on and discussion of one’s morality. In certain situations, one’s moral routines do not function self-evidently anymore, for instance in a moral dilemma or through the confrontation with another perspective. This leads to moral doubt or disagreement, which can serve as stimulus for moral learning and moral development [33]. In those situations, moral routines are challenged as they fail in providing guidance for action. As Cook states, “if we are open to questioning our ethical habits and beliefs, we will be open to discovering mistakes and new ethical truths” ([6], p368). In these situations, the awareness might arise that the habits that, up to that time, have guided us efficiently are no longer able to provide us with sufficient guidance for practice ([22], p. 61). These impasses require the ability and willingness to look at one’s value system as such, and venture into that of other stakeholders. One needs to become aware of one’s moral routines and engage in a process of moral inquiry in which one’s moral presuppositions are reconsidered in light of the situation at stake. This implies that morality is “*dynamic”* and is subject of change ([8, 19, 43], p. 95–7 & p.144–5).

Pragmatism and hermeneutics, both emphasize that morality is *contextual*. Moral inquiry requires interaction with the environment and with the people involved in the situation at stake. As a consequence, ethics develops in action [3, 33]. Contextual factors are morally relevant and this implies that what is right cannot be determined without taking into account the specific characteristics of the situation. In line with this, from a hermeneutic point of view, it can be argued that our concepts and actions acquire their meaning within actual practices. The same action can be interpreted differently by different people (influenced by their own specific moral background), and even by one person in different situations. “Ethics is therefore ‘intimately concerned with the timely, the local, the particular and the contingent (e.g. what should I do *now*, in *this* situation, given *these* circumstances, facing *this* particular person, at *this* time).” ([1], p.245).

Since ethics always starts from a specific here, *at this* moment *by these* practitioners, rules, norms and values are not “dogmas” ([8], p.96) but “instruments” ([22], p. 58) which acquire their meaning within the practices in which they are used. “Conceptions, theories and systems of thought are always open to development through use. […] They are tools. As in the case of all tools, their value resides not in themselves but in their capacity to work, shown in the consequences of their use.” ([8], p145). Values, norms and rules require interpretation and application to the situation. To clarify this dynamic; Gadamer refers to the notion of jurisprudence, in which the application of legal rules to concrete situations leads to new insights [14]. In order to apply a rule or norm, a person needs experience and expertise. In line with this, organizational literature emphasizes that blindly following a rule is typically a mistake of a novice. Part of becoming proficient in a trait is knowing when the situation requires to depart from a rule [7].

According to hermeneutics and pragmatism, there is no 'God perspective' [5] on a situation, there are only interpretations from specific perspectives. This has consequences for CES. First, as one perspective is not closer to ‘the truth’ than another perspective, ethical reflection is often about contrasting your own perspective with that of another and learning from this. Second, interaction with the world is a fundamental source of knowledge, and therefore sources like bioethical literature, expert opinion or normative theories are not a priori more important than experiential knowledge of the situation or a specific perspective from a stakeholder. No knowledge source (just because of its type) is epistemologically better than another. All knowledge sources should be taken seriously. They all represent a specific angle and interpretation of an event.

Given that all perspectives are limited, moral learning requires opening up to the perspectives of others [47, 48]. According to hermeneutics, this takes the form of a dialogue, which can result in a fusion of horizons. People are always constrained by the limits of their situatedness at a specific time and place (their horizon), but through dialogue, a fusion of horizons can take place in which one’s perspective is enriched with another perspective [14]. This process does not lead to a 'God Perspective', universal or objective knowledge as human learning is essentially finite. The situatedness of human action implies that we cannot reason about what is ethically right in an abstract manner, without taking the particularities from a certain situation into account [8, 9]. Instead, our ethical considerations are heavily influenced and constrained by our surroundings at a particular moment, for instance the health care infrastructure, the physical surroundings, the possible courses of actions, the rules and regulations, one’s own competencies, etc.

Learning is not only dependent on opening up to the perspectives of others, but also contains a physical and bodily dimension that has often gone unnoticed by dominantly cognitive understandings of learning [17, 27, 34, 38]. Reading a textbook, following a lecture, or having a cognitive understanding is often not sufficient for an actual change in behavior. Inspired by and expanding on Dewey, Schön emphasized that practical, intuitive and embodied knowledge is far more influential in our actual behavior in certain contexts than intellectual, cognitive and rational knowledge [17, 38]. Schön put forward *an epistemology of practice*, arguing that *within practices* specific tacit and bodily knowledge is generated (knowing-in-action) [26]. Although it must be stressed that this is not the only form of knowledge development and learning according to Schön, this kind of knowledge is often not recognized but very influential for our behavior and difficult to put into words.

To sum up, our theoretical perspective is based on the following assumptions. First, we regard morality and ethics as contextual and dynamic. Second, we believe that morality is mostly invisible and develops by growing up in and moving through the world. Third, we assume that ethical reflection starts when moral routines are challenged. People start reflecting on their morality, i.e. doing ethics, when confronted with a practical problem which forces them to reconsider their moral habits. Fourth, we emphasize that moral concepts and rules acquire their meaning within concrete practices and that this requires interpretation; i.e. what does this rule or principle require of me in this situation? How can it guide and inform my action? Fifth, we acknowledge that different individual perspectives and kinds of knowledge sources are needed to reach a meaningful interpretation of a situation, and can be fused in a process of dialogue. Finally, for actual change to happen, the reflection should not start nor remain at a cognitive level. In the next paragraph we will consider what this theoretical background means for the development of CES.

### Case study

The case study concerns CES for the Centre of Expertise on Gender Dysphoria (CEGD) of the Amsterdam University Medical Center, Location VUmc in the Netherlands. LH and BM were mostly involved in providing and developing the CES. GW occasionally provided CES and was involved in peer debriefing. AWK contributed with developing the integrative approach to CES from the CEGD perspective and GI contributed to the theoretical reflections and their implications for integrative CES.

The CEGD provides health care to individuals who experience Gender Dysphoria (GD) and desire gender affirmative treatment. GD refers to the distress as a result of an incongruence between one’s gender identity and assigned gender at birth [16]. Gender affirmative care consists of a trajectory of different phases [4], depending on the individual’s treatment wishes. Client centered transgender health care requires multidisciplinary involvement of psychologists, psychiatrists, endocrinologists, plastic surgeons, urologists, pediatricians, nurse specialists and gynecologists that closely cooperate.

In the following we give a brief overview of the CES activities we developed for the CEGD. We describe these activities and the lessons we learned in more details elsewhere [18].

At the yearly policy meetings of the CEGD, about 80–100 professionals of all involved disciplines, meet up for one full day or afternoon and discuss the latest developments surrounding their care. In 2013, during one of these meetings, in which both the adult and youth care providers were present, the first CES initiative was organized for the CEGD team and consisted of facilitating moral case deliberations (MCDs). MCD is a structured dialogue, with a facilitator on a concrete case that is experienced by participants as morally troublesome [41]. In the context of a grant for empirical-ethical research on moral controversies in gender treatment, we evaluated these MCDs [44]. To follow up on the results of this evaluation study and create more ownership of CES, we set up a small steering group for CES at the CEGD. The members of the steering group were LH and BM as CES staff, and AWK and other members of the management team of the CEGD, who played a formal role at the policy level. At these steering group meetings both the form, content, and implementation of CES were discussed as well as the follow-up of the CES activities within care processes and treatment policy of the CEGD.

Together with the steering group six new and innovative CES activities were developed.

1. For 6 months, the CES staff took part in the weekly multidisciplinary meetings organized by the clinic’s staff. The CEGD hired LH and BM for their presence.
2. The CES staff collaborated in several CEGD related education activities (on both bachelor and master level).
3. LH, BM and some members of the CEGD set up a research project to map the moral dilemmas that were experienced by the staff of the CEGD. CES staff and CEGD staff together published an article about these dilemmas [15].
4. Based on the study of moral dilemmas, and a selection of important moral dilemmas by the CEGD team, thematic log- and working groups on specific moral topics were created.
5. Besides MCD, creative methods were used to discuss moral issues at the regular policy meeting of the team, e.g. online voting systems regarding normative statements about shared decision making in transgender care.
6. An interactive workshop, a so called ‘couch discussion’ was developed to address ethical issues in gender affirmative care at five international conferences, three on gender affirmative care and two on CES. The CES support team and the CEGD team together presented both the content and the developmental process of the co-created CES, and facilitated a discussion on pervasive moral issues in an international context.

## Results

Against the theoretical background described above and by reflecting upon a case study of providing CES at the Centre of Expertise on Gender Dysphoria (CEGD) (see above), we formulated five key characteristics for an integrative approach to CES:

1. Positioning CES more within care practices;
2. Involving new perspectives;
3. Creating co-ownership of CES;
4. Paying attention to follow up;
5. Developing innovative CES activities through an emerging design.

These key characteristics are based on iterative processes in which both theory and practice played a role. We did not derive them from theory and then applied them to the CES practice or vice versa. Instead, they are based on a continuous interaction between practical experience and theoretical reflection. In other words, there was no predefined idea beforehand of what integrative CES would or should look like, we rather derived the characteristics of integrative CES by first experiencing and developing them in practice and then reflecting on our experience relating them to theory, and finally giving them a name.

### Positioning CES more within care practices

This first key characteristic refers to the location of CES. Often CES activities are separate from the place where care is provided and organized. For instance, both an ethics committee and MCD involve meetings that are usually detached from the busy atmosphere of everyday care. Both on request of the steering group (see methods) and inspired by the theoretical framework above, we experimented with CES activities that take place *within the actual care practices*. We describe three CES activities we developed in order to offer ethics support within daily work. We use the theoretical framework described above to deepen our understanding of our experiences during these activities.

First, LH and BM joined the multidisciplinary treatment teams as CES professionals. In these weekly meetings, treatment decisions were made. During these meetings, LH and BM made observatory notes and asked clarifying questions (‘e.g. Why did you choose to not treat this patient?’ or ‘How does this relate to argument X which was just mentioned before?’) or more normative questions (e.g. ‘Why do you think this is the right decision?’).^1^ Through asking these questions, LH and BM aimed to make the existing implicit moral frameworks more explicit and thereby visible and subject of discussion for the team. LH and BM also tried to connect themes, viewpoints, arguments and conclusions from earlier CES activities with the issues that were discussed in the multidisciplinary treatment teams.

Second, LH and BM and some members of the CEGD performed ethnographic research on ethical dilemmas experienced by the CEGD team [15]. A student researcher observed the multidisciplinary meetings and analyzed the MCDs. Through observation, the ethical dilemmas were derived from clinical practice; this included those dilemmas emerged within the multidisciplinary meetings which were not immediately recognized by the CEGD team as ethical dilemmas. The findings were grouped in different ethical themes and were presented to the team during the CEGD policy meeting. This made the team members aware of the ethical issues they were unaware of until then. The staff remarked that the way we structured our observations into ethical themes was very different from the one usually carried out by one of the members of the CEGD team; the themes were structured according to an ethical perspective instead of a medical perspective.

Third, we experimented with writing an ‘ethics log’ (or ethics diary) on two topics during every day work processes or observations (i.e. ethical dilemmas concerning ‘fertility’ and ‘life style’ (e.g. Body Mass Index and smoking). In order to create more focus within the large amount of ethical issues and to guarantee a stronger harvest for clinical practice and policy, four members of the CEGD team and two CES staff members volunteered to keep a ‘shared ethics logbook’ in which they wrote down all ethical issues around topics selected by the team (i.e. how often it occurred, the kind of ethical dilemmas, other questions, were/how it was discussed, what kind of arguments were used, what kind of discussion took place, what the final decision and actions of the team were). The CES staff developed a first framework or structure of the ‘ethics logbook’ which was adjusted together with the CEGD team during several working group meetings (i.e. what and how to log topics). During CEGD team meetings both the CEGD and CES loggers presented their preliminary findings. The loggers chose one specific topic to discuss with the whole team in four interactive workshop sessions.

In all three examples the CES interventions focused on addressing or reconstructing ethical issues which emerged during actual work. Throughout these initiatives the ethical dimension of the everyday practice was made explicit, instead of steering the reflection on a pre-determined ethical issue or theme. This helped the CEGD team members to create a meaningful connection between ethical reflection and practice; the ethical deliberation was not relegated to a separate CES activity but became part of daily work. This direct interrelatedness between ethical reflection and practice was triggered merely by the presence of the CES staff, the student researcher or the loggers. Even if they did not say anything, the CEGD team recognized them as CES staff focusing on the ethical dimension of their work. For example, in one of the multidisciplinary team meetings, somebody said: ‘*This is really a dilemma*’ and then looked at the specific ethics staff member. At another moment, a participant remarked: ‘*Perhaps you should note this down, since there is really an ethical issue here*’. Also, more often CEGD team member referred to specific MCD sessions in which the topic at hand had been discussed before.

In line with our theoretical background, we consider an important task of CES to make the invisible moral frameworks visible, and thereby subject of reflection for the care workers. We found that organizing CES activities *within care practices* can be valuable since it urges the CES professionals to help the team in revealing the ethical dimension of every day issues that were previously interpreted as merely factual, medical or were even completely unnoticed by the team. As a result, the ethics work was no longer limited to the moments in which the CEGD team participated in specific CES activities.

Yet, we also encountered some challenges when positioning CES more within care practices. In the first place, time for reflection appeared to be limited during actual work processes. For example, the ‘interventions’ from the CES staff during multidisciplinary meetings were restricted to short questions; there was no time for a more thorough discussion of the issues or arguments. Within care practices health care workers are pressed for time and often do not experience the room and space to reflect on their own moral framework. The CES professionals were able to point out ethical issues, in everyday decision making that had gone unnoticed by the care workers themselves, but the time to further discuss or reflect on these ethical issues had to be organized on separate occasion.

To conclude, the first characteristic, positioning CES more within care practices, raised awareness on moral routines and implicit moral reasoning. This enabled the team to understand, discuss and adjust them (when deemed necessary). By reflecting on automatic judgements during actual work processes, professionals uncover normative presuppositions, values and norms within their judgements, decisions or discussions. From a dialogical perspective, developing a new understanding of a disagreement or confusion within a team, with the patient or among stakeholders, can help the health care team to realize why certain choices are difficult, and provide them with a new vocabulary to describe and talk about the ethical dimension of their practice. This enables professionals to voice their questions, jointly reflect on their daily routines and habits and foster reflection about their own presuppositions in light of the characteristics of the situation they face every day.

### Involving new perspectives

The second key characteristic of an integrative approach to CES is the involvement of new perspectives. From our theoretical framework above, follows that there is no ‘God perspective’ from which one can determine what is the right thing to do. For integrative CES this means that one should actively try to include new, unexamined perspectives within the ethical reflection, and encourage the team to integrate these perspectives in the consideration of the right thing to do. A new perspective can be provided by a colleague with whom one strongly disagrees or a stakeholder who was not part of the discussion before. New perspective on an existing situation can also be brought in by considering an ethical theory or by inviting CES staff to be present and observe care practices. As described above, moral learning entails opening up to multiple perspectives. We describe two examples in which the team was challenged to consider new perspectives on a certain ethical issue.

First, MCDs were organized as a way to bring several perspectives together and stimulate moral learning by a dialogue between these perspectives [41, 47]. Within the MCDs LH and BM stimulated interdisciplinary exchanges, by dividing the attendees of the policy days into smaller groups (of about 12 people each) aiming for as much variety of representatives of disciplines as possible within each MCD. This provided new insights and more understanding between the disciplines. Within MCD, health care workers are challenged to reflect themselves on what they consider the right thing to do in a specific case, within the general framework of rules, regulation, research evidence and quality standards. The stakeholders are either present at the MCD or represented by participants of the MCD who are encouraged by the facilitator to place themselves in the shoes of the stakeholders.

A lot of moral dilemmas re-emerged about the question on what was the best age to suppress puberty by means of puberty blockers (GNRH antagonists). Care for youngsters (age between 10 and 12) that wish for a medical transition can be roughly divided in two phases. After a first diagnostic phase, the initiation of puberty suppression enables a lengthening of the diagnostic phase that offers extra time to reflect upon gender identity development and treatment wishes without the development of secondary sex characteristics. Puberty suppression is reversible.

In the Netherlands, in general, puberty suppression can be initiated from the age of 12 after extensive diagnoses and meeting specific criteria (e.g. Tanner stadium)., while androgens and estrogens can be prescribed from the age of 15, also meeting certain criteria. These two phases, first the start of puberty suppression at 12 and then androgens or estrogens from 15, seem relatively straightforward. But in daily practice a lot of questions and discussion arise around the question; what is the right time to start puberty suppression?

For instance, some youngsters, especially those assigned females at birth, reach puberty before age 12. The biological development of puberty is independent of the calendar age and is in clinical terms expressed in Tanner stage. The team struggled with the question; should we start puberty suppression younger than 12? Should we make an exception especially for biological girls? First, there was discussion in the team whether the Tanner stage or calendar age should be taken as leading for prescribing puberty suppressing hormones. After carefully deliberating this issue, they decided for the Tanner stage. But then, moral questions and disagreements started to emerge about ‘what is then ‘the right’ Tanner stage? The team discussed a number of these cases in interdisciplinary MCDs. One insight they gained is that their opinion also differed according to the nature and responsibility of their respective disciplines. Some professionals wanted to start earlier because the less physiological characteristics of the biological sex, the more the youngster would eventually pass as the preferred gender. However, some psychologists emphasized the importance of the youngster being very sure of the trajectory (since the trajectory has some very serious consequences like infertility). They also referred to some evidence that in a proportion of adolescents, gender dysphoric feelings may diminish when a youngster reaches puberty. As a consequence, some caregivers in some cases preferred to wait a little bit longer, until upcoming puberty (but before reaching full puberty), to see how the youngster would react to the hormones of their biological sex (to be really sure the youngster still wants this and not regrets the decision later). The MCDs resulted in more understanding in these various perspectives, improving the awareness of individual factors that play a role in this deliberation and a leading towards a more shared approach concerning the Tanner stage at which to start puberty suppression.

During the MCDs participants were asked to investigate values and norms which are important for the stakeholders in the case, especially those who are not present during the MCD [41]. This exercise made explicit the moral dimension of the case and led to a better understanding regarding their own judgement and the judgments of other participants. Through dialogue with others, participants provided a new description of the case at stake by making differences explicit and contrasts visible. Moreover, they were asked to link their values to concrete norms for action thereby reflecting on concrete rules of action which follow from their values.

This exercise is valuable from both a hermeneutic and pragmatists perspective [21, 47]. Through dialogue with others, who might have different ideas, professionals acquire more understanding about their own presuppositions. They are encouraged to engage in a dialogue and to be curious about others’ opinions instead of aiming to convince each-other. Dialogue is hermeneutically used to foster a joint learning process which enables participants to reflect upon their own moral intuitions. Moreover, as pragmatism stresses, the first step towards the resolution of a (moral) issue is providing a new and enlarged description of it ([35], p.78), which requires including different interpretations.

This example shows that the CES activity of organizing MCD can help with involving various perspectives in a moral consideration, and can stimulate the exchange and joint learning process between disciplines. Organizing MCDs can be a valuable part of an integrative approach to CES, since it encourages moral learning by reflection on one’s moral routines through opening up new perspectives.

A second example of involving a new perspective concerns the introduction of theoretical concepts from ethical theory. LH and BM observed that ‘shared decision making’ was a pervasive moral theme during MCDs and treatment meetings. To discuss this theme with the whole team, LH and BM used five normative statements on moral responsibilities within shared decision making, partly based on literature [42] and partly based on their experiences within the CES activities.^2^

We discussed these statements on two different occasions; at an international conference regarding transgender care 48 [20] and at a team meeting of the CEGD. We used voting software and asked the attendees a) what was their position with respect to *the actual* and the *ideal situation* of shared decision making in transgender care, and b) which value was decisive for their ideal position. The opinions and values of the attendees were collected anonymously and projected in real time on a screen. This allowed participants to engage in a conversation about the results of the voting. Both the attendants at the international conference and the team at the team meeting remarked that the five normative statements enabled them to discuss a pervasive and difficult ethical theme in a more structured and more constructive way.

This exercise shows that theoretical concepts can provide a new perspective on, or clarify an ethical theme. As part of an integrative approach to CES, CES staff can search for metaphors, insights or concepts from (bioethical) literature, that support health care professionals in clarifying their ethical dilemmas, or provide new vocabulary to describe and discuss the ethical dimension of a certain situation. When this is done successfully, the participants can learn to better understand the specific situation and also other similar ones, thereby improving their ability to act on their heightened awareness. Connecting theoretical concepts to specific experiences of practitioners transforms the theoretical concepts into tools which are immediately useful for practice.

It is impossible to determine beforehand what kind of concepts resonate well with the experience of care practitioners. As elaborated by Dewey, ethics is not a set of rules which can be applied in all situations [10]. Principles and theories do not apply to all situations; context specificity does not allow to determine beforehand what kind of concepts resonate adequately with the health care practitioners. The abstract notions, need to support the practitioners in ‘making sense’ of their own practices.

We also experienced challenges with the involvement of new perspectives, e.g. organizing patient involvement as a new perspective was particularly challenging. We know from the literature that patient involvement requires good preparation and specific care and attention for both patients and caregivers [45]. Within the case study, we had some experiences with involving clients in CES. At the WPATH, EPATH and a public evening at the CEGD we simulated an MCD in which also transgender people participated. The first experiences were positive; they allowed participants to enter in a dialogue (rather than a debate in which opposing groups of stakeholders try to convince each other) and encouraged more understanding into the perspectives of others. We lacked the resources and time up until now, to follow up on this and organize the involvement of patients in a proper and careful way.

To conclude, we consider the effort to engage new perspectives in various ways an important part of an integrative approach to CES. These other perspectives can be the view of a coworker who is in disagreement, or the perspective of a patient that may have gone unheard before. This goal can be achieved by inviting new stakeholders or to organize MCDs involving different professional disciplines. It may also mean organizing observational research or inviting CES staff to participate in the daily activities. Furthermore, engaging new perspectives can also be achieved by introducing theoretical concepts, principles or insights from (bioethical) literature, which then need to be applied to the concrete situation.

### Creating co-ownership of CES

A third key characteristic of an integrative approach to CES is working towards co-ownership regarding both use and follow-up of the CES, and the further development of CES. We experienced that co-developing the form and structure of the CES together with the team helps in making the CES activities actually respond to the needs for CES and to the care structure of the team. This requires the CEGD team to have an active, ‘ownership-kind’ of feeling towards CES instead of a more passive attitude.

Often, CES is interpreted as the expertise and responsibility of the CES professional. This implies that CES professionals are regarded as guardians of the ethics climate or that facilitating and organizing ethics activities is seen as the role of CES staff and not of the health care team, which remains passive. For instance, when ethics staff is responsible for organizing MCD, this can be a barrier for the uptake of the insights from this MCD session by the health care team. In our project co-ownership gradually took place. The founding of a steering group for CES (see characteristic 2.4. for more detail), played an important role in this. In the steering group a continuous (informal) evaluation of the current CES methods took place. Based on the evaluations, new strategies for action and new policies were developed and then communicated to the whole team. This led to continuously co-developing and experimenting with new CES methods (see also 2.5). Here, we describe two other activities which were helpful in creating co-ownership of CES.

First, an evaluation study about the MCDs at the policy meetings played a fundamental role in establishing a co-ownership of CES [44]. In addition to the fact that the facilitators (LH and BM) got in-depth information about the needs for CES and the team’s experience of specific MCDs, this also encouraged the team members of the CEGD to actively put the kind of CES they needed on the agenda. The design of the evaluation study also contributed to this. The evaluation was a mixed methods study, combining questionnaires with interviews and focus groups. During the focus groups, the question whether MCD addressed the needs sufficiently, what could be improved and whether other CES activities would be more useful was discussed extensively. So, in line with responsive evaluation methodology [2], instead of evaluating CES on predefined outcomes, the desired outcomes of the CES activities were discussed with the team. This made the team aware that they ‘had a say’ and even ‘should have a say’ in the kind of CES they received and helped creating co-ownership of the CES activities.

Second, we experienced that undertaking certain activities together, like presenting at conferences, jointly developing and teaching working groups and lectures for students and co-authoring papers, fostered trust, informal relationships, collegiality and a sense of co-ownership of CES. During the joint preparation of these sessions, activities and co-authoring of papers, the meaning of the CES activities for the team was discussed within the broader international context of both CES and gender affirmative care. For instance, LH and BM gained in-depth insights about the ethical controversies and international debates within gender affirmative care, and the position of the Dutch CEGD in this. Likewise, CEGD professionals gained more understanding of specific kinds of CES, different views on CES and the position of the Department of Medical Humanities in this matter.

We consider a sense of co-ownership of the CES activities an important aspect of integrative CES, both practically and theoretically. As pragmatist and hermeneutic ethics argue, ethics is always contextual and develops within practices. This also applies to CES itself. The form, method and way of organizing CES should be adjusted and evaluated within practices in order to provide the right kind of support (see also 2.5.). This entails including stakeholders in the way the CES is organized and with what kind of method the CES is provided, in sum – striving towards a co-ownership of CES.

Creating a sense of co-ownership of CES does come with challenges. An important challenge is time. The first contact with the CEGD was established in 2013, when an MCD was organized during a policy day. It took time to gradually intensify the contact and familiarize with the way of working and culture. It can take years of joint learning experiences and building trust to achieve true co-ownership of CES. A second challenge was to determine the level of activity required of the CES professionals to strive towards co-ownership. At the beginning of CES, the ownership was not experienced by the CEGD team, so the CES staff put extra effort in the organization and continuation of CES, leading to the risk that the CEDG would become more passive (i.e. if someone else does all the work, there is no incentive to organize something yourself). After a while, the CEGD team started to take the lead more with specific requests for CES, prompting the CES staff to become more passive and thinking about innovative ways (see 2.5.) to be able to meet the request for CES. In sum, the ‘right amount of activity and responsibility’ to strive for co-ownership of CES differs over time, and requires reflection and responsiveness to new circumstances.

To conclude, we experienced that a participatory and responsive research design for evaluation research, in which the stakeholders are challenged to not only evaluate CES on predefined outcomes, but express the outcomes they themselves would want and the roles they could play in improving the usefulness of CES, can help towards more co-ownership of CES. Secondly, undertaking and jointly developing certain activities, like presenting a session on a conference, teaching or co-authoring papers, works well to create more trust and informal relationships and it also helps to explicate to each other the meaning of certain CES activities within a broader context. We experienced that these activities helped creating more co-ownership of CES and embedding it in clinical practice.

### Paying attention to follow up

A fourth characteristic of an integrative approach to CES concerns extra attention for the follow up of CES activities in order to actually contribute to improvement of clinical practices. Within CES one reflects on what is considered to be good care for a specific situation, or for a certain ethical issue. A cognitive understanding of the right thing to do is often not sufficient for actual change in behavior. It is important to follow up on new insights and translate them into action. We describe two examples in which we stimulated follow-up from the CES activities in clinical practice.

The evaluation study of the MCDs we conducted showed that we needed to pay more attention to the follow up of the MCDs [44]. To follow up on the results of this evaluation study, we set up a small steering group for CES at CEGD. The members of the steering group were LH and BM as CES staff, AWK and other members of the management team of the CEGD who played a formal role at the policy level. At these steering group meetings, the form, content, and implementation of CES was discussed as well as the follow-up of the CES activities within care processes and treatment policy of the CEGD. Besides fostering follow-up actions, the steering group also played an important role in creating more co-ownership (characteristic 2. and 3.).

To stimulate follow-up, an inventory of follow-up points was made at the end of each MCD. The facilitators asked the participants which insights/remarks/outcomes of the MCD they felt needed to be taken up. In regular meetings with the steering group these points were also discussed. For instance, during one MCD it was remarked that the team lacked information and expertise to handle religious doubts about gender affirmative care from a Muslim perspective. The steering group decided to invite an expert to give a lecture on this topic at the next CEGD policy day. This way, the learning points of the MCDs and other CES activities became integrated within the actual care process of the team (on a micro-level, concerning a treatment decision and meso-policy level developing a local protocol or fostering the knowledge of the team members).

Another example concerned the follow up of the study mapping all ethical issues [15]. At a policy meeting of the CEGD team, the overview of all ethical issues was presented in order to perform a member check with respect to the findings of the study. In addition to this, the presentation of the findings was used to ask the CEGD team members which ethical issue they would like to prioritize and who would like to be involved in the follow-up. As discussed above, the CEGD team decided to continue to work with two ethical themes. Subsequently, four CEGD team members volunteered to become a member of a working group, and to use the ethics logbook. Based on the notes and reports of the logbooks, a specific policy group was established, chaired by a CES staff member, in order to develop a new policy for the ethical issue.

A challenge we experienced concerns the responsibility for follow-ups. We deliberately prevented emerging of a situation in which CES professionals are responsible for the CES activities and the team of the CEGD for the follow up in their care, and aimed for a more diffuse division of responsibilities (see also 2.3. co-ownership). The CES professionals put topics on the agenda, actively questioning whether they were followed up on, and even thinking along into how certain changes could best be implemented in the care processes. The steering group increasingly collaborated in defining which kind of CES activity would benefit them the most at a particular moment. The absence of a clear division of responsibility was positive, in that it created a joint effort to foster CES as well as improve practice. Yet, this also led to discussion and sometimes a lack of clarity about who is responsible for what. We continuously reflected on this and made it a topic for discussion.

To conclude, as emphasized in our theoretical framework, just having a cognitive understanding of what the right thing to do is, is not sufficient for actual change to happen. Actual changes in morality require the development of a new way of doing things, and concrete changes in the working culture or context (e.g. how the work is organized). To prevent ethical dilemmas from recurring, more systemic change is required (ranging from extra education to a change in infrastructure). This requires cooperation between the CES professionals and the team, and joint work on making explicit what are the follow-up practical implications of moral insights and concrete decisions emerged during the CES activities.

### Developing innovative CES activities through an emerging design

A fifth characteristic of integrative CES is that it requires an emerging design and cannot be planned from the start to the end. This implies that both the type of CES activities themselves and the co-ownership of CES cannot be fully preordained, but evolve during the process. In order to respond to changing CES needs, a creative, flexible and participatory attitude of both CES staff and CEGD staff is essential. Ideally new CES activities are developed together with the health care professionals (co-ownership 2.4.). As mentioned in our theoretical framework, we learn from our experiences, and continually readjust and redirect existing CES activities based on the previous experiences and on the continuously changing context.

The collaboration and development of new and creative CES activities evolved gradually over time (since 2013 and ongoing). In the steering group, but also in other constellations, we developed a number of innovative CES activities, which worked well for the team in the sense that the methods enabled them to discuss certain moral themes and returning topics. For instance, the ethics logbooks and joining the treatment meetings (see above) were innovative CES activities arising from joint learning experiences and reflection on what was needed at a particular time. Both examples aimed at bridging more regular CES activities outside the working place with the actual professional behavior in clinical practice, i.e. moving towards clinical practice (see 2.1.). Also, an emerging design does not imply that certain initiated CES activities are expected to go on indefinitely. Hence, an emerging design also implies that certain CES activities do stop after a while because they do not longer serve their original purpose. Whereas the co-creation and co-ownership framework of collaboration leading to the development of integrative CES activities is currently still in place, and has become a shared responsibility of the CES team and health care professionals, the resulting activities have evolved according to the needs of the team and the inputs coming from the CES team. Sometimes the need for a certain CES activity has passed, or the momentum has seized due to different circumstances. An emerging design requires a continuous moving along with new circumstances and co-creating innovative CES activities.

In line with the theoretical framework, an emerging design implies that we need to learn from our experiences and evaluate our concepts and methods for integrative CES within practices. We learned from our experiences and continually readjusted and redirected the CES activities based on what worked and didn’t work. For instance, the initial structure of the ethics logbook appeared to be too complex. Based on the working group meetings with the CEGD loggers, we simplified the logbook and we planned more additional meetings or talks with the CEGD loggers to discuss and write down the ethical issues within specific cases/experiences/situations for the logbook.

A challenge resulting from the emerging design is that it can give rise to questions within a hospital structure that requires project plans, goals and definitions of outcomes for instance with regard to financing (e.g. there is only money to do 6 MCDs a year). When you cannot specify beforehand exactly which CES activities you will perform, it is difficult to calculate a budget for the CES staff and the CEGD staff. There was no previously set out work plan or project plan for the CES for the CEGD. It was part of the larger structure of offering different kinds of CES for the whole hospital. Within this larger hospital-based structure there was explicit room for variations to the needs of the local teams/wards or departments. Furthermore, the CEGD team was able and willing to provide additional funding for trying out new ideas. This is one way to address the challenge of keeping flexibility within a larger structure which often requires more planning.

To conclude, an important part of integrative CES makes it impossible to all CES activities ahead, but leave room for flexibility, innovativeness and a continuous readjusting and fine-tuning of the CES activities towards the needs of a particular team or the aims that we are striving for; i.e. an emerging design. This is in line with our theoretical framework in which it is emphasized that our concepts and theories acquire their meaning within practices and continuously need to be readjusted based on the experiences within practices.

## Discussion

In this paper we described an integrative approach to CES by elaborating on five key characteristics. These key characteristics are based on our experiences in a case study and our theoretical reflection on ethical theories, especially pragmatism, hermeneutics, and educational and organizational studies. Integrative CES aims at positioning the CES activity closer to the regular work processes (both physically with respect to the location of CES and methodologically, i.e. presenting the ethics activities as connected to the work activities). Integrative CES also puts additional effort in creating co-ownership of CES and in strengthening the follow-up of conclusions, insights and actions derived from the CES activities. Finally, it emphasizes the importance of flexibility and developing innovative CES activities in an emerging design.

The integrative approach to CES we put forward in this paper can be compared to *integrated* CES developed in the US and “the hub and spokes strategy” developed in Canada [13, 28]). Integrated CES as described by Fox and colleagues provides a comprehensive, systemic and programmatic approach to CES based on ethics consultation (level of decisions and actions), preventive ethics (level of systems and processes) and ethical leadership (level of environment and culture) [13]. The ‘hub and spokes’ strategy provides an alternative model in which the ethics support is decentralized by training health care workers as local ethics recourse leaders (the spokes). The health care ethicist (the hub) “focuses to enhance awareness, knowledge, and skills by building and supporting ethics capacity” and training the spokes. ([28], p. 258).

A similarity between the integrated ethics approach, the hub and spokes strategy, and our integrative approach to CES is the aim to increase the impact of CES within a health care organization by organizing CES in a new and comprehensive way. Another similarity is the combination of both cased based support and attention for the policy level, to prevent ethical dilemmas from happening again (preventive ethics )[13].

The main difference is that the integrative approach to CES put forward in this paper is not programmatic, like the integrated approach and, to a lesser degree, the hub and spokes strategy. The notions of emerging design and development of innovative CES activities refer to a more dynamic approach. The idea of Integrative CES is that CES professionals and health care professionals continuously evaluate and re-adjust the CES towards the needs of that moment. This can be both the needs as experienced by the team and the needs that the CES professionals feel need to be addressed. We chose the verb *integrative* instead of *integrated* CES, to emphasize the inherent active and dynamic character of this approach, while at the same time keeping the emphasis on integration of CES within care practices and the larger organization. The hub and spokes strategy also emphasis this need for flexibility in the sense that the spokes can provide CES adapted to the local context [28]. A further difference is that the integrative approach to CES and the hub and spokes approach target the whole hospital, whereas our integrative CES approach focuses on a specific team. We need to further research if this kind of radical co-ownership of CES is also possible on a hospital level.

An integrative approach to CES seems to be a way to meet the challenges for current CES mentioned in the introduction of this paper. First, in our case study there was no shortage of issues for reflection and deliberation. In fact, the steering group had to discuss which themes should be given priority give the large number of topics put forward, in light of limited resources (in money and time of professionals). Second, the impact of CES on the care practices and the chance of uptake of the outcomes of CES appears to be higher than in more traditional forms of CES, because of the alignment of CES with the needs of a team, the emphasis on follow-up of the CES activities and the commitment to co-ownership. The emphasis on co-ownership stimulates the health care team to express the outcomes they themselves would want and the roles they could play in achieving these outcomes (instead of evaluating CES on predefined outcomes). In future studies, it is important to actually evaluate integrative CES activities in the light of the concrete outcomes of integrative CES.

With regard to the question of whether a CES professional should be an in- or outsider, our experience with integrative CES seems to indicate that a middle position is possible and effective. Because LH and BM were often present, they felt like insiders, and were treated as such; yet, they remained CES professionals, and did not have a standard role in the team. In this sense, our approach shows that the distinction between in- an outsider roles is not straightforward, and that the merits of both roles should be determined in view of the concrete situation [39]. Following Aristotelian virtue ethics, one might argue that it is important to prevent the extremes of full immersion on the one hand, and external criticism on the other hand, and to be sensitive for the right middle in concrete situations. Also, it helps to discuss these tensions explicitly internally and with the health care team.

We also experienced that the middle position between insider and outsider provided a good basis for critically examining existing elements of health care practice, together with the team. Since we were regarded as partners, we were able to highlight moral issues that had gone unnoticed by the team. Indeed, as we have argued, a crucial role of the CES professionals is to make certain moral routines visible and subject of discussion, by fostering new perspectives on a situation. The case study indicates that trust is crucial for critical reflection. Trusting each other does not mean merely being nice. We experienced that the trust of the health care team allowed us to ask critical questions. We also experienced that a dialogical approach to ethical reflection enabled stakeholders to actually reflect upon what has being said (instead of defending themselves).

A strength of our methodology in developing an integrative CES approach is the combination of practical experience and theoretical reflection. The key characteristics we have described are the outcome of a process of reflection, linking practical experience to theoretical concepts, resulting in a better understanding of both theory and practice. Moreover, we made our theoretical background explicit, in order to be transparent about the presuppositions concerning the way we perform CES, the CES activities we prefer, and the perspective from which we analyze the results [36, 37]. This opens our viewpoint up for further discussion.

The five key characteristics can guide evaluation and further professionalization of CES in general and of a local CES practice. However, we should be aware that the characteristics should not be treated as new dogmas or rules that apply for all CES practices. In line with our theoretical framework, good CES should be determined and assessed in on ongoing dialogue, within local CES practices, with the relevant stakeholders.

## Conclusion

In this paper we described five key characteristics of an integrative approach to CES. 1. Positioning CES more within care practices, 2. Involving new perspectives, 3. Creating co-ownership of CES, 4. Paying attention to follow up, and 5. Developing innovative CES activities through an emerging design. They are based on theoretical reflections (pragmatism, hermeneutics and organizational and educational sciences) and our experiences in a case study in which we together with a team of health care professionals in transgender affirmative care developed a CES approach fitted to the specific practice. Our experience has been that integrative CES requires efforts, both of the clinical ethicists and of the team of health care professionals, but is also rewarding, in that it is shared work, directly related to regular work processes and resulting in new ways of dealing with the ethical dimensions of daily care.

## Data Availability

Since this is a more theoretical oriented paper, no additional data are available in a database. Supporting documents from the CES activities we provided could be made available upon request (in Dutch).

## Abbreviations

CES: Clinical Ethics Support
CEGD: Centre of Expertise on Gender Dysphoria

## Footnotes

1 We reflected on our experiences in this new role in more detail in another paper [18]

2 Five levels of decision making:1.The caregiver should decide, taking into account what is known about the treatment2.The caregiver should decide, taking into account the preferences of the patient/client3.The caregiver and patient/client should decide together, based on equality4.The patient/client should decide, taking into account the preferences of the caregiver5.The patient/client should decide, taking into account what the patient/client knows about the treatment
